# Causes of Death

**Published:** 1884-01

**Authors:** 


### CAUSE OF DEATH.

Medical science is much indebted to the able researches of *Wundt*, in one of these, the important induction is drawn that "The proximate cause of death is Asphyxia," that is to say, "*a man dies for want of breath*," and science has found it out.

There is a nerve which comes from the brain, grows out of it, as it were, and in coming from the head, it divides into two branches, one of which goes to the stomach, the other to the throat and lungs. If you cut off the stomach branch, there is no digestion; if you divide the lung branch there is no breathing. If you injure one branch, that injury, if kept in continuance, affects the other branch, hence it is, that dyspeptic people have throat ail, sooner or later; hence it is, that such persons dwindle away, and if not cured, fall into *a decline*. The consumptive may eat a great deal; and he has a good appetite to the last day of his life, but his food does not seem to afford nourishment, because the stomach branch of the nerve has lost its power, hence he eats, but it gives him no strength, he has not the strength to breathe without an effort, and that effort he has not power to make except at intervals, hence consumptives breathe short and quick; and shorter and quicker to the last struggle. Consumptive people do not die for want of lungs, as is generally supposed. A man can live an age with half of all his lungs in full operation, and live in considerable health, too. General Jackson had lost a third of his lungs, as his autopsy indicated twenty years before his death. Most consumptives die long, very long, before half their lungs are gone; and why? simply for *want of breath!* for want of bodily power to fill the lungs they have, to their full, of pure air. To have bodily strength, we must have a good digestion, and good digestion will give bodily strength under all circumstances, hence to cure a consumptive, that is, to arrest the further progress of lung decay, and enable him to live on what lungs he has left, the man must be made to digest substantial meat and bread, the most healthfully nourishing of all human edibles—as a means of enabling him to draw in pure air. Therefore, we are impelled to the conclusion, and it is one of world-wide significance, that there are no means of arresting the progress of consumptive disease in any case, except by increasing the capabilities of the stomach of food digestion, *to the end* that the lungs be empowered thereby to draw in and use a larger amount of pure air, that very air which the Almighty, in his wisdom, has made to be food for the lungs.

By a section, a cutting off, of this nerve of which we have been speaking, the *Pneumogastric*, *Wundt* found that it required more time and more strength to draw a sufficient breath, the breathing then became slower, the quantity of air inspired gradually diminished, the body grew colder, the lungs became clogged, and the victim died. Therefore, reader, if you wish to be a "*well man*" perfect your digestion, perfect your good breathing.

